# Monosomal karyotype and chromosome 17p loss or *TP53* mutations in decitabine-treated patients with acute myeloid leukemia

**DOI:** 10.1007/s00277-020-04082-7

**Published:** 2020-06-06

**Authors:** Heiko Becker, Dietmar Pfeifer, Gabriele Ihorst, Milena Pantic, Julius Wehrle, Björn H. Rüter, Lars Bullinger, Björn Hackanson, Ulrich Germing, Andrea Kuendgen, Uwe Platzbecker, Konstanze Döhner, Arnold Ganser, Anne Hagemeijer, Pierre W. Wijermans, Hartmut Döhner, Justus Duyster, Michael Lübbert

**Affiliations:** 1 Department of Medicine I, Medical Center – University of Freiburg, Faculty of Medicine, University of Freiburg, Hugstetter Straße 55, 79106 Freiburg, Germany; 2 German Cancer Consortium (DKTK) partner site, Freiburg, Germany; 3 Clinical Trials Unit, Medical Center – University of Freiburg, Faculty of Medicine, University of Freiburg, Freiburg, Germany; 4 Department of Internal Medicine III, University Hospital of Ulm, Ulm, Germany; 5 Charité University Medicine, Berlin, Germany; 6 Interdisciplinary Cancer Center Augsburg, Augsburg, Germany; 7 Dept. of Hematology, Oncology and Clincal Immunology, University Clinic Düsseldorf, Heinrich-Heine University Düsseldorf, Düsseldorf, Germany; 8 University Hospital Dresden, Dresden, Germany; 9 University Hospital Leipzig, Leipzig, Germany; 10 Department of Hematology, Hemostasis, Oncology and Stem Cell Transplantation, Hannover Medical School, Hannover, Germany; 11 Department of Human Genetics, University of Leuven, Leuven, Belgium; 12 Haga Ziekenhuis, The Hague, The Netherlands

**Keywords:** Decitabine, Acute myeloid leukemia, AML, Mutations, TP53, Monosomy

## Abstract

*TP53* aberrations reportedly predict favorable responses to decitabine (DAC) in acute myeloid leukemia (AML). We evaluated clinical features and outcomes associated with chromosome 17p loss or *TP53* gene mutations in older, unfit DAC-treated AML patients in a phase II trial. Of 178 patients, 25 had loss of 17p in metaphase cytogenetics; 24 of these had a complex (CK+) and 21 a monosomal karyotype (MK+). In analyses in all patients and restricted to CK+ and MK+ patients, 17p loss tended to associate with higher rates of complete remission (CR), partial remission (PR), or antileukemic effect (ALE). Despite favorable response rates, there was no significant OS difference between patients with or without loss of 17p in the entire cohort or in the CK+ and MK+ cohort. *TP53* mutations were identified in eight of 45 patients with material available. Five of the eight *TP53*-mutated patients had 17p loss. *TP53*-mutated patients had similar rates of CR/PR/ALE but shorter OS than those with *TP53* wild type (*P* = 0.036). Moreover, patients with a subclone based on mutation data had shorter OS than those without (*P* = 0.05); only one patient with *TP53*-mutated AML had a subclone. In conclusion, 17p loss conferred a favorable impact on response rates, even among CK+ and MK+ patients that however could not be maintained. The effect of *TP53* mutations appeared to be different; however, patient numbers were low. Future research needs to further dissect the impact of the various *TP53* aberrations in HMA-based combination therapies. The limited duration of favorable responses to HMA treatment in adverse-risk genetics AML should prompt physicians to advance allografting for eligible patients in a timely fashion.

**Electronic supplementary material:**

The online version of this article (10.1007/s00277-020-04082-7) contains supplementary material, which is available to authorized users.

## Introduction

The hypomethylating agents (HMA) decitabine (DAC) and azacitidine (AZA) are a standard of care in AML and higher risk MDS patients not eligible for intensive treatment. While dynamic features, such as early platelet response [1], can be used to estimate eventual treatment response, no pre-treatment markers are in routine clinical use.

Several studies reported associations between outcomes of patients with MDS or AML receiving HMAs and genetic aberrations, DNA methylation, mRNA or microRNA expression, or other markers (e.g., HbF) [2–14]. Among these, we described that patients with AML or MDS and a monosomal karyotype (MK+) benefitted from DAC treatment, particularly when multiple monosomies were present. [3, 9, 15] The MK+ status is closely associated with the presence of a complex karyotype (CK+) [15], and MK+ and CK+ are associated with mutations in *TP53* [16–19]. In turn, *TP53* mutations have also been recently reported to be predictive for response in patients with MDS or AML treated with DAC [11, 14]. Moreover, among patients with chromosome 17p aberrations, those treated with AZA tended to have a better overall survival (OS) than those treated with conventional care regimens [16].

Here, we evaluated the genetic and clinical characteristics associated with loss of chromosome 17p or gene mutations affecting *TP53* in older, unfit AML patients treated with DAC within a phase II trial.

## Patients and methods

### Patients and treatment

Patients were enrolled onto the phase II trial 00331 (German Clinical Trials Registry DRKS00000069), the results of which have been previously reported [3]. Briefly, 227 patients with AML (by French-American-British classification), who were ineligible for intensive chemotherapy, were treated with DAC (15 mg/m^2^ every 8 h for 3 consecutive days, total dose of 135 mg/m^2^, every 6 weeks). In case of an antileukemic effect (ALE) or stable disease (SD) after course 1, administration of the second course of DAC was followed by all-*trans* retinoic acid (ATRA; 45 mg/m^2^/day) for 28 days. Patients with complete remission (CR), partial remission (PR), or ALE after completion of 4 courses were eligible to receive maintenance treatment with DAC at 20 mg/m^2^/day (for 3 consecutive days, every 6–8 weeks). Bone marrow aspirates were performed after courses 1, 2, and 4. Morphology was centrally reviewed. The following response definitions were applied [3]: CR: BM blasts < 5%, platelets > 100 × 10^9^/L, white blood cells (WBC) > 1.5 × 10^9^/L, and no extramedullary leukemia. PR: BM blasts 5–25%, platelets > 100 × 10^9^/L, WBC > 1.5 × 10^9^/L, and no clinical or imaging evidence of leukemia; or BM blasts < 5%, platelet count < 100 × 10^9^/L, WBC < 1.5 × 10^9^/L. ALE: > 25% reduction of BM blasts relative to the initial blast percentage but not enough to fulfill the criteria for a PR. The study was approved by the institutional review boards of each center. All patients had given written informed consent for collection and use of data and specimens. All procedures followed were in accordance with the ethical standards of the responsible committee and with the Helsinki Declaration.

### Cytogenetics and gene mutations

Metaphase karyotypes were centrally reviewed and CK+ and MK+ status assigned as previously described [3, 15]. MK+ required presence of a single autosomal monosomy and a structural aberration, or two or more autosomal monosomies [15]. Loss of 17p was evaluated based on the available karyotype data.

Data on mutations in *DNMT3A* and *NPM1* and *FLT3*-internal tandem duplications (ITD) had been previously reported [17]. For the present study, bone marrow (*n* = 27) and peripheral blood (*n* = 18) samples of 45 patients were analyzed using the Illumina TruSight Myeloid Sequencing Panel (covering 54 genes relevant in myeloid neoplasms) for library preparation and an Illumina MiSeq device for sequencing. Variants located in introns, synonymous variants, and known single nucleotide polymorphisms were excluded. Variants had to feature a variant allele frequency (VAF) of > 5% for missense variants, or had to be hot spot mutations or mutations known to be present in the given patient, or had to be large insertions or deletions. Variants had to be covered by > 100 reads, and the variant had to be observed in > 10 reads. Four of six amplicons covering *CEBPA* only gave insufficient reads for analysis, thus *CEBPA* mutations may be underestimated. The genetic data were used to derive the clonal architecture (detailed in the supplemental).

### Statistical analyses

CR, PR, ALE, SD, progressive disease (PD), early death (ED). and OS (time from start of treatment to death) were defined as previously described. [3] All patients who had received at least one dose of DAC were included in the analysis. The Fisher’s exact and Wilcoxon rank sum tests were used to compare categorical or continuous variables, respectively. Estimated probabilities of OS were calculated using the Kaplan-Meier method. Group differences were assessed using the log-rank test and univariate Cox proportional hazards models.

## Results

### Association of loss of 17p with pre-treatment characteristics and outcomes

As previously published, [3] cytogenetic data were available for 177 patients; 120 patients had clonal cytogenetic aberrations. Of these, 25 patients were identified to have loss of 17p; 24 of them were CK+, and 21 were MK+ (Table 1).

**Table 1:** Clinical characteristics of AML patients with or without loss of 17p

	Loss of 17p (*n* = 25)	No loss of 17p (*n* = 152)	*P* value
Age, years median (range)	71 (61–82)	72 (56–86)	0.49
Sex, *n* (%) female	11 (44%)	51 (34%)	0.37
Prior MDS, *n* (%) yes	10 (40%)	88 (58%)	0.13
WBC, × 10^9^/L median (range)	2.7 (0.5–83.3)	5.8 (0.6–86.2)	0.05
Platelets, × 10^9^/L median (range)	56 (12–207)	36 (3–894)	0.08
Hemoglobin, g/dl median (range)	8.6 (5.8–11.3)	9.0 (1.4–13.0)	0.44
LDH, U/L median (range)	383 (180–4081)	279 (121–2261)	0.06
PB blasts, % median (range)	11 (0–63)	15 (0–96)	0.53
BM blasts, % median (range)	61 (10–100)	50 (18–100)	0.21
CK status, *n* (%) CK+ CK−	24 (96%) 1 (4%)	28 (18%) 124 (82%)	< 0.001
MK status, *n* (%) MK+ MK−	21 (84%) 4 (16%)	17 (11%) 135 (89%)	< 0.001
DAC courses, *n* median (range)	2 (1–12)	2 (1–23)	0.66

*MDS* myelodysplastic syndromes, *nd* not determined, *FAB* French-American-British classification, *WBC* white blood cells, *LDH* lactate dehydrogenase, *PB* peripheral blood, *BM* bone marrow, *CK* complex karyotype, *MK* monosomal karyotype, *DAC* decitabine

**Fig. 1:**
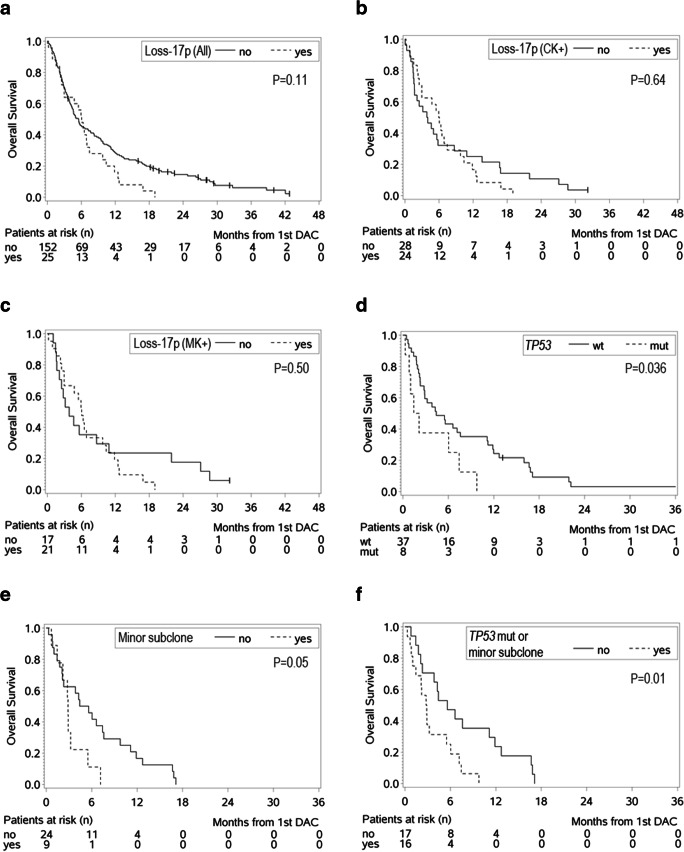
Overall survival according to **a-c** the presence or absence of loss of 17p among **a** all patients with cytogenetic information, **b** patients with CK+ and **c** patients with MK+, and overall survival according to **d** the presence or absence of a *TP53* mutation among all patients with samples subjected to panel sequencing (corresponding COX model: HR 2.31, 95% CI 1.03–5.16, *P* = 0.041), **e** the presence or absence of minor subclones among patients with available data (corresponding COX model: HR 2.29, 95% CI 0.98–5.39, *P* = 0.056), and **f** the presence of a *TP53* mutation or a minor subclone or absence of both among patients with available data (corresponding COX model: HR 2.63, 95% CI 1.20–5.79, *P* = 0.016)

We evaluated the outcome of patients with loss of 17p compared with those without in the entire cohort of patients with cytogenetic data and in the subgroups of CK+ or MK+ patients (Table 2, Fig. 1a–c). Patients with loss of 17p overall tended to have favorable response rates in comparison with patients without loss of 17p (CR/PR/ALE vs SD/PD/ED, *P* = 0.08). This was also true when analyses were conducted only among patients with CK+ (*P* = 0.01) or MK+ (*P* = 0.05). However, despite these favorable response rates and although the median OS was longer for patients with loss of 17p especially in the CK+ and MK+ cohort, there was no significant difference in the OS between patients with or without loss of 17p in the entire cohort or in the CK+ and MK+ cohort, as the OS curves crossed shortly after the 6-month mark.

**Table 2:** Comparison of response rates and overall survival in various genetic groups

	CR	PR	ALE	SD	PD	ED	CR/PR/ALE *n* (%)	CR/PR/ALE vs SD/PD/ED *P* value	Median OS (months)	*P* value
Loss of 17p (*n* = 25)	6	4	8	3	1	3	18 (72%)	–	6.0	–
No loss of 17p (*n* = 152)*	20	20	38	38	17	16	78 (52%)	0.08	5.5	0.11
CK+/loss of 17p (*n* = 24)	6	4	7	3	1	3	17 (71%)	–	6.0	
CK+/no loss of 17p (*n* = 28)	2	4	4	7	6	5	10 (36%)	0.01	3.9	0.64
MK+/loss of 17p (*n* = 21)	6	4	6	2	1	2	16 (76%)	–	6.0	–
MK+/no loss of 17p (*n* = 17)	2	2	3	6	3	1	7 (41%)	0.05	3.8	0.50
*TP53* mut (*n* = 8)	0	1	2	1	1	3	3 (38%)	–	1.8	–
*TP53* wt** (*n* = 37)	0	6	12	10	4	4	18 (50%)	0.70	4.4	0.036

*CR* complete remission, *PR* partial remission, *ALE* antileukemic effect, *SD* stable disease, *PD* progressive disease, *ED* early death, *OS* overall survival, *CK* complex karyotype, *MK* monosomal karyotype

*In 3 patients, best response was not evaluable

**In 1 patient, best response was not evaluable

Of the patients with cytogenetic data, 77 had received ATRA in addition to DAC, 49 of them over the entire planned period of 4 weeks during course 2. Only six of the 49 patients had a loss of 17p. The low number of patients and the bias regarding the selection of patients receiving ATRA precluded further analyses.

### Gene mutation profiles and clonal architecture

**Fig. 2:**
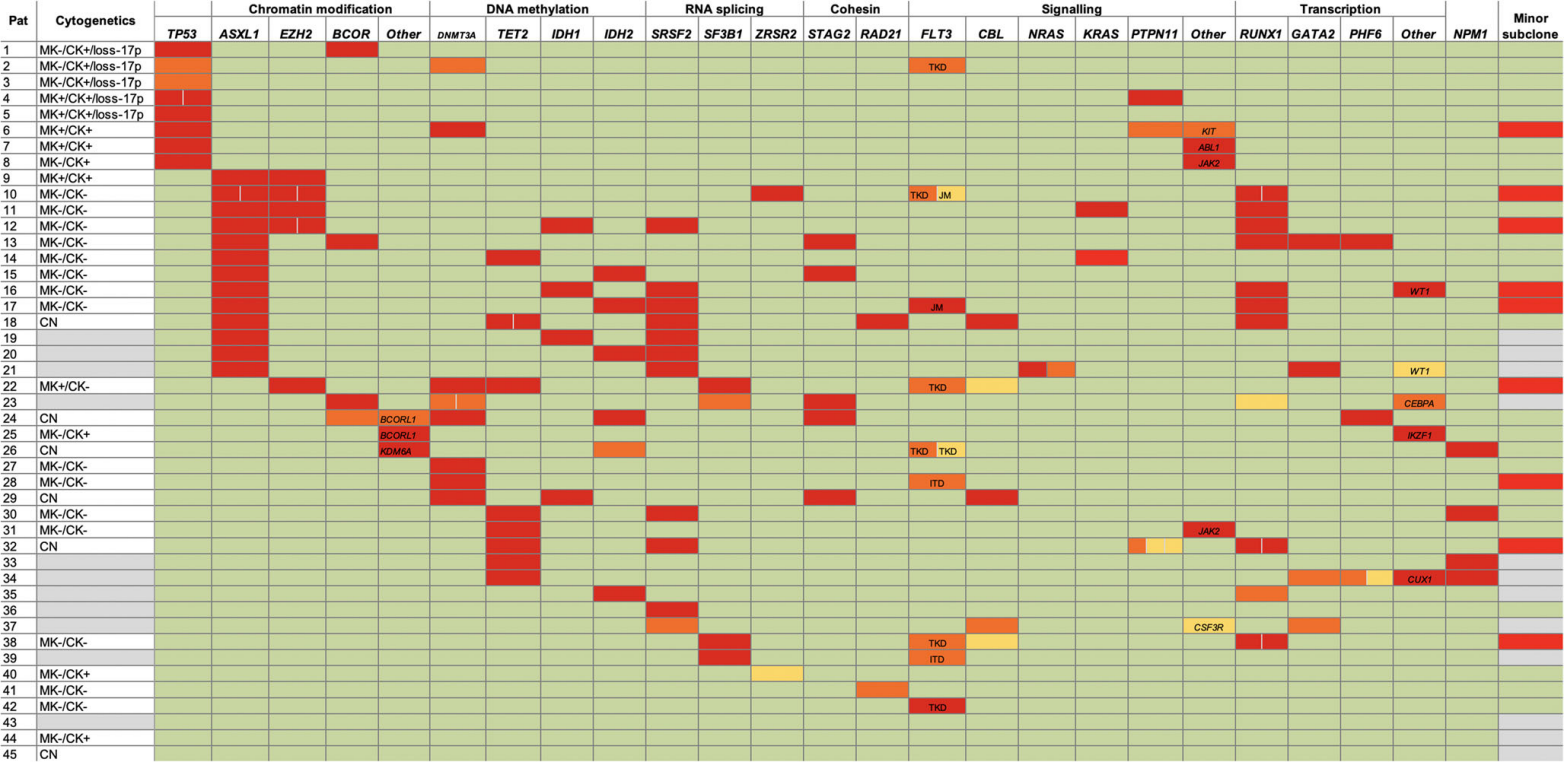
Genetics in AML patients analyzed via panel sequencing. Each line represents a patient, and each column contains the genetic information as indicated. Patients are ordered by the presence of a *TP53* mutation and/or loss of 17p, and then by the mutation status of the remaining genes according to their order in the columns. In case of mutated genes, the variant allele frequency (VAF) is indicated: VAF > 25%, red; 11–25%, orange; ≤ 10%, light orange. If a gene harbored more than one mutation in a patient, the VAF of each mutation is provided. Wild-type sequence is color-coded in green. Rarely mutated genes are summarized in a column called *other* and the gene name is specified in the cell. For mutations in *FLT3*, the localization is specified: ITD, internal tandem duplication in the juxtamembrane (JM) domain; JM, point mutations in the JM domain; TKD, point mutations in tyrosine kinase domain. The presence of a minor subclone is indicated in red, its absence in green. Missing data are color-coded in gray

Among the 45 patients with samples available for sequencing, the median age was 75 years (range, 63–83), and 27 (60%) had AML secondary to MDS. We identified a median of three mutations (range, 0–9) or three mutated genes (range, 0–6) per patient, respectively; nine patients had more than one mutation in a given gene, and in three (7%) patients, no mutations were identified (Fig. 2, Supplemental Table S2). Most frequently mutated genes were *ASXL1* (29% of patients), *RUNX1* (24%), *SRSF2* (24%), *IDH1* (9%) or *IDH2* (13%), *TET2* (18%), and *TP53* (18%).

Based on the sequencing and cytogenetic data, the clonal architecture could be derived in 33 patients (Fig. 2, Supplemental Table S2). In nine patients, one or more minor subclones (defined through mutations present in a cell fraction that was > 20% smaller than the major clone) were present; while in the remaining 24 patients, no subclones in addition to the major clone could be identified.

### Genetic and clonal features of the *TP53* mutations

The *TP53* mutations were located in exons 4 (*n* = 2), 5 (*n* = 3), 7 (*n* = 3), and 10 (*n* = 1) (Supplemental Table S2); one patient had two mutations. Three *TP53* mutations were truncating, the remaining were missense substitutions.

Five patients with a *TP53* mutation also had a cytogenetic loss of 17p. In one of these patients, the VAF of the *TP53* mutation indicated the loss of the *TP53* wild-type allele (i.e., VAF > 60%); one other patient with loss of 17p had two mutations in *TP53*. One patient with a *TP53* mutation with a VAF > 60% had no chromosome 17 aberration by metaphase karyotyping. *TP53*-mutated AMLs more often were CK+ (*P* < 0.001), MK+ (*P* = 0.02), or harbored a loss of 17p (*P* < 0.001) than *TP53* wild-type AML (Table 3).

**Table 3:** Characteristics of patients with samples analyzed via panel sequencing according to the *TP53* mutation status

	*TP53* mut (*n* = 8)	*TP53* wt (*n* = 37)	*P* value
Age, years median (range)	71 (66–77)	77 (63–83)	0.01
Sex, *n* (%) female	2 (25%)	16 (43%)	0.45
Prior MDS, *n* (%) yes	7 (88%)	20 (54%)	0.12
FAB, *n*			n.d.
M0	0	4	
M1	2	8	
M2	6	5	
M4	0	7	
M5	0	2	
M6	2	4	
WBC, × 10^9^/L median (range)	4.05 (0.90–11.30)	4.80 (0.50–35.70)	0.72
Platelets, × 10^9^/L median (range)	34 (3–137)	42 (9–324)	0.61
Hemoglobin, g/dl median (range)	8.2 (6.5–9.3)	8.9 (5.0–10.8)	0.19
LDH, U/L median (range)	320 (203–894)	281 (134–2216)	0.19
PB blasts, % median (range)	28 (0–63)	20 (0–90)	0.57
BM blasts, % median (range)	47 (10–81)	69 (21–95)	0.17
CK status, *n* (%)			< 0.001
CK+	8 (100%)	4 (15%)	
CK−	0	22 (85%)	
nd	0	11	
MK status, n (%)			0.02
MK+	4 (50%)	2 (8%)	
MK−	4 (50%)	24 (92%)	
nd	0	12	
Loss of 17p status, *n* (%)			< 0.001
Loss of 17p	5 (63%)	0	
No loss of 17p	3 (38%)	25 (100%)	
nd	0	11	
DAC courses, *n* median (range)	1 (1–6)	2 (1–11)	0.22

*MDS* myelodysplastic syndromes, *nd* not determined, *FAB* French-American-British classification, *WBC* white blood cells, *LDH* lactate dehydrogenase, *PB* peripheral blood, *BM* bone marrow, *CK* complex karyotype, *MK* monosomal karyotype, *DAC* decitabine

The *TP53* mutations were all present in the major AML clone of the respective patient. Only one patient (13%) with a *TP53*-mutated AML had a minor subclone, while 8 (32%) of the patients with *TP53* wild-type AML did. Patients with a *TP53* mutation harbored a median of only one additional mutation (range, 0–4).

### Association of *TP53* mutations with clinical features and outcome

Compared with *TP53* wild type, patients with *TP53* mutations were younger (*P* = 0.01; median, 71 vs 77 years); AML with *TP53* mutation tended to more often develop from antecedent MDS (*P* = 0.12) (Table 3).

In the outcome comparisons between AML patients with mutated or wild-type *TP53*, there were no differences in the response rates, but patients with *TP53* mutations had a shorter OS than those with wild-type *TP53* (*P* = 0.036) (Table 2, Fig. 1d).

Twenty-four of the patients with sequenced samples had received ATRA. Of these, only three harbored a *TP53* mutation, which precluded outcome analyses.

### Association between other mutations and outcome

Other markers previously reported to be associated with outcomes in DAC-treated patients are mutations in *SRSF2*, [11] *DNMT3A*, [2, 17] *TET2*, [7] *IDH1*, or *IDH2*. [8, 14] We found no differences in response rates or OS between patients with or without mutations in these genes or in at least one RNA splicing gene (data not shown). Moreover, there were no differences in response rates and OS between patients with ≤ 3 mutated genes and those with > 3 mutated genes (Supplemental Table S3).

However, the presence of subclones was associated with a shorter OS in comparison with their absence (*P* = 0.05) (Fig. 1e, Supplemental Table S3). Only one of the 9 patients with a minor subclone also harbored a *TP53* mutation. Since both a *TP53* mutation and presence of a minor subclone were associated with shorter OS, we combined patients with at least one of these features into one group and compared them with the remaining. Compared with patients with *TP53* wild type and absence of a minor subclone, those with a *TP53* mutation or a minor subclone expectedly had shorter OS (*P* = 0.01); no differences in the response rates were observed (Fig. 1f, Supplemental Table S3).

## Discussion

HMAs have become a standard of care in AML patients not eligible for intensive chemotherapy. Chromosomal or molecular aberrations of *TP53* are likely central in the investigation of markers and biological pathways associated with the response to HMAs [5, 7, 10–12, 14, 16, 18, 19]. Thus, we sought to investigate the impact of loss of 17p and *TP53* mutations in our phase II trial 00331 in which older unfit AML patients were treated with 3-day DAC.

We observed that patients with a loss of 17p tended to have higher rates for CR/PR/ALE both in analyses including all patients and in those restricted to CK+ and MK+ patients. Among CK+ and MK+ patients, patients with loss of 17p also had longer median OS, but this favorable course could not be maintained over time. Patients with *TP53*-mutated AML had similar rates of CR/PR/ALE but shorter OS than those with wild-type *TP53* (*P* = 0.036).

Published data on the impact of chromosome 17p aberrations on response to HMA treatment are scarce. Nazha et al. [20] observed no difference in the response rates according to chromosome 17 aberrations in MK+ and CK+ patients treated with HMAs. In an explorative retrospective analysis of the AZA-AML-001 study, patients with chromosome 17p aberrations had a strong trend for better OS when treated with AZA as compared with conventional care regimens (mainly low-dose cytarabine) [16].

The impact of *TP53* mutations (or expression) on outcomes in HMA-treated MDS or AML patients has been assessed in several studies and yielded heterogeneous results. [7, 10–12, 14, 16, 19] Welch et al. [11] observed in patients with AML or MDS that achievement of CR was more frequent in patients with a *TP53* mutation. Moreover, in contrast to the poor OS of *TP53*-mutated AML patients after standard induction, there was no OS difference according to *TP53* mutation status in patients receiving DAC. In the aforementioned analysis of the AZA-AML-001 study, patients with *TP53*-mutated AML had strong trends for improved OS when treated with AZA compared with alternative therapies [16]. However, in studies among MDS patients treated with DAC or AZA, *TP53* mutations had no impact on response rates but were associated with shorter response duration and/or OS [7, 10]. In another report on patients with MDS (mostly with blast excess) who received DAC, most patients with *TP53* mutations achieved a CR, but they still had inferior OS [14].

In MDS, mono-allelic *TP53* mutations associate with more favorable disease features (including less frequent complex karyotype and better OS) than multi-hit *TP53* mutations [21]. The role of *TP53* mutations under consideration of their allelic state remains to be established. Welch et al. [11] described that two-thirds of the patients with *TP53* mutations potentially had both alleles affected. In our study, 5 patients fulfilled the criteria of a *TP53* multi-hit mutation (i.e., multiple gene mutations or gene mutation plus genomic loss) according to Bernard et al. [21]. The low patient numbers precluded meaningful outcome analyses. Hopefully, future studies will be able to decipher the impact of the allelic state of *TP53* and its impact in response to DAC.

Considering our results and those reported by others, it currently remains elusive whether the *TP53* aberrations or rather associated genetic features may confer sensitivity to HMAs. As observed in the present study, *TP53* mutations and chromosome 17p aberrations coincide with CK+ and MK+ [22–26]; and for trial 00331, which was subject to the present study, we previously reported that MK+ patients had higher response rates and similar OS compared with MK− patients [3]. Similar results have been reported by Wierzbowska et al. [27] from a post hoc analysis of the phase 3 DACO-016 trial in AML and by our group from the phase 3 EORTC trial 06011 in MDS [9, 28]. In the study by Welch et al. [11], almost all patients with *TP53* mutations had unfavorable cytogenetics, and achieving a CR was more frequent in patients with unfavorable than those with intermediate or favorable cytogenetics. In the study by Chang et al. [14], almost all *TP53*-mutated patients who achieved a CR were CK+ or had monosomies.

Welch et al. [11] suggested that a variable response of *TP53*-mutated AML to DAC may be due to the presence of *TP53* mutations in subclones instead of the major clone. We observed no superior response to DAC, although *TP53* mutations were all present in the major clone, and despite other features supporting their disease-driving effect, i.e. *TP53*-mutated AML only rarely harbored a minor subclone and had a low number of additional mutations [7]. However, we did observe that patients with a minor subclone had shorter OS than those without, although only one of the nine patients with a minor subclone also harbored a *TP53* mutation.

The heterogeneity in the reports on associations between *TP53* aberrations and response to HMAs may be due to weaknesses that are variably shared by the studies, including the present study. First, analyses are often based on relatively small patient numbers [11, 14, 16]. Second, if provided, the information on 17p loss normally stems from conventional cytogenetics, although the (presumably lost) *TP53* allele may be present in unidentified chromosome material. [16, 29] Third, cohorts variably comprise MDS or AML patients or both, although DAC may have higher efficacy in patients with higher blast counts [30, 31]. Fourth, there is the heterogeneity in treatment. In several studies, patients treated with DAC or AZA were combined into one group [7, 10], or patients were included who received DAC combined with another agent [2, 3, 7, 10]. Moreover, DAC was administered according to different protocols. The majority of the patients received DAC according to the 5-day protocol (total of 100 mg/m^2^ over 5 days) [10, 14]. In the study by Welch et al. [11], the majority of patients received DAC according to the 10-day protocol (total 200 mg/m^2^ over 10 days). Patients in our present study received DAC according to the 3-day protocol (total of 135 mg/m^2^ over 3 days; every 6 weeks), in part of the patients followed by a reduced dosage maintenance phase. Moreover, in the present study, the patients with loss of 17p or *TP53* mutation had received only a median of 2 (range, 1–12) or 1 (range, 1–6) DAC courses, while several courses are normally required to achieve best response.

While the clinical observation of the (counter-intuitive) response to HMAs in adverse genetics AML/MDS is more and more accepted within the clinical community, the underlying mechanism of the interaction between hypomethylating activity and these genotypes is still unresolved. Monosomal chromosomal regions may preferentially attract epigenetic silencing [32, 33], providing a particularly sensitive target to DNMT inhibition. Despite the present lack of a conclusive model of this interaction, clinicians need to be aware that the responses, while surprisingly frequent, are often short-lived. Hence, they can also be quite deceptive, by raising unfounded optimism regarding their duration. Thus, patients with adverse genetics who are eligible for allografting should transition to this curative treatment in a timely manner, i.e., before HMA resistance sets in.

In summary, within the specifications of the patient cohort studied, loss of 17p was associated with trends for higher DAC response rates, both within the entire cohort and among patients with CK+ or MK+ AML. Patients with a *TP53* mutation achieved similar response rates as patients with wild-type *TP53*, but had a shorter OS. Our data further support the potential applicability of *TP53* aberrations as predictor for HMA treatment, and emphasize a possible role for subclonal mutations in this regard. Isolated *TP53* mutation analyses apparently are not sufficient for prediction of HMA response. Cytogenetic analysis remains standard and allows for evaluation of MK+ status and 17p loss. The landscape of HMA-based therapy is changing, and favorable responses in adverse genetics patients are also observed when these drugs are combined with the BCL-2 inhibitor venetoclax [34] or all-*trans* retinoic acid [35]. Hence, the impact of the different types of adverse cytogenetics and *TP53* alterations (e.g., cytogenetic and molecular genetic mono- or bi-allelic loss) on outcome after HMA combination studies will be of great interest.

## Electronic supplementary material

ESM 1 (PDF 448 kb).
