# Supplementary material for: Monosomal karyotype and chromosome 17p loss or TP53 mutations in decitabine-treated patients with acute myeloid leukemia
Source: Ann Hematol. 2020 Jun 6;99(7):1551–60. doi: 10.1007/s00277-020-04082-7 (PMC7316846; doi:10.1007/s00277-020-04082-7)
Supplement: Supplementary file 1 — (PDF 448 kb). [file 277_2020_4082_MOESM1_ESM.pdf]

Supplemental Information

Heiko Becker, Dietmar Pfeifer, Gabriele Ihorst, et al. Monosomal karyotype and chromosome 17p loss or TP53 mutations in decitabine-treated patients with acute myeloid leukemia

Supplemental Methods

Derivation of the genetic clonal architecture

The proportion of cells harboring distinct mutations was estimated by multiplying the VAF by 2 (to a maximum of 100%) assuming a heterozygous state of the mutation. For mutations with a VAF >60%, affected by a deletion or monosomy (as concluded from cytogenetics) or located on chr X in male patients a heterozygous state could not be assumed and, thus, the VAF was considered to be similar to the number of affected cells. For mutations located on chromosomes affected by a trisomy, the clonal architecture was evaluated by multiplying the VAF by 1.5 and 3, under the assumption that the mutated or wild-type allele was gained, respectively. Only samples with encompassing cytogenetic data or cases with only one mutation identified were considered. Cytogenetic aberrations were not considered clone-defining events, due to the generally low number and the processing of cells analyzed by metaphase cytogenetics. Mutations that were present in a proportion of cells that was by >20% smaller than the mutation with the highest VAF were considered to have been acquired later during disease course. Mutations with ≤20% difference in the proportion of cells affected were concluded to be present in the same clone. Mutations in minor subclones were present in a proportion of cells that was by >20% different than the mutation with the lowest VAF in the major clone.

Prediction of DNA copy number variations from NGS data

We attempted to retrieve data on chr17 copy number variations (CMV) based on the sequencing data in patients without cytogenetic information and without a *TP53* gene mutation using the CNVPanelizer (Oliveira C and Wolf, T; R package version 1.4.0), but we were not able to reliably derive such data. Briefly, the amplicons representing a gene according to the manufacturer's TruSight Myeloid Targeted Regions BED File were used as input for the CNVPanelizer algorithm. PCR duplicates were not removed and bootstrapping and background methods were repeated 10,000 times. In order to evaluate this approach for the identification of CNVs based on the available NGS data, data from samples with normal karyotype (based on metaphase cytogenetics) were compared with data from samples with -17. The results obtained by the algorithm based on the NGS data did not match the data from the metaphase cytogenetics. Thus, we concluded that the density of data points of the available NGS data is not sufficient to reliably predict CNV.

Supplemental Table S1. Karyotypes with a loss of 17p aberration

Pat	Karyotype	CK	MK
1*	46,XY,del(20)(q1?3.1)[2]/44~45,X,-Y,del(5)(q31),der(6)?t(1;6)(q25;q21),der(16;17)(q10;q10),add(17)(p1?),del(20)(q1?3.1),+mar,inc[cp18]	+	-
2*	55-56,XY,+Y,+r(4),dic(5;17)(q13;p11),+8,+9,+11,+13,+14,+18,+20,+der(22)t(11;22)(q14;p11)[5]	+	-
3*	48,XY,der(2)t(1;2)(p11;p25),der(3;12)(q10;q10),dic(5;17)(q11;p11),+8,+21 [8]/46,XY,der(2)t(1;2)(p11;p26),der(2;12)(q10;q10),dic(5;17)(q11;p11),der(15)ins(15;22)(q22;q?q?)[3]/46,XY [1]	+	-
4*	46,XX,del(5)(q13q33)[2]/44,XX,del(5)(q13q33),-7,-16,-17,+mar [26]/46,XX [2]	+	+
5*	44-46,XY,del(2)(p13),der(3)(q21),del(5)(q13q33),del(7)(q22q32),del(13)(q14q22),-15,-16,-17,-18,del(20)(q11),+?21,+1-3mar,inc[cp17]/46,XY[5]	+	+
6	52,XX,+del(1)(q24),del(5)(q13q33),del(7)(q21q32),+11,+13,+14,+16,-17,+21,+22 [7]	+	+
7	46,XX,del(5)(q13q33),der(16)del(16)(p11)del(16)(q22),-17,der(20)t(17;20)(q11;q11),+der(20) [17]	+	+
8	42,XX,inv(3)(q21q26.2),del(5)(q13q33),-7,-11,-14,add(14)(p11),-17,der(17;18)(p10;q10),-18,+mar [16]	+	+
9	~41,XY,add(1)(q32),-3,-5,-7,-12,del(16)(q22),-17,-18,del(20)(q11q13),+mar, dmin (matrix CGH)	+	+
10	39-41(XY),-Y,del(4)(q11),der(3)t(3;5)(p21;?)t(5;17),der(5)t(5;17)(q31;q11),-8,der(11)t(3;11)(p21;q23),der(14;15;21;21),+16,-17,del(20)(q11),+21[cp15]	+	+
11	47-48,XX,del(3)(p11p14),-5,del(6)(p22),+del(6)(p22),-7,+8,+11,add(17)(p11),-18,add(22)(q11),+1-2mar [20]	+	+
12	45,XY,del(3)(p21),-5,-7,del(10)(q22),der(12;22)(q10;q10),+13,del(17)(p11) [15]	+	+
13	42,XY,der(?)t(3;?)(q21;?),del(5)(q11q22),der(8),ish 8q22(ETO-),trp(11)(q13q23),-16,-17,del(20)(q11),der(21),inc [cp7]/46,XY [1]	+	+
14	46,XX,del(5)(q13q31)[9]/46,XX,del(5)(q13q31),del(17)(p11)[11]	-	-
15	46,XY,der(5;17)(p10;q10),-17,-18,add(19)(p13),+21,+mar [23]	+	+
16	45,XX,del(5)(q13q33),-7,-17,+mar [12]/45,XX,del(5)(q13q33),-7,i(8)(q1 0),-17,+mar [7]	+	+
17	45-46,XX,-2,-4,-5,add(7)(q?22),add(12)(p1?),-?14,-?15,-17,+5-6mar,inc[cp6]	+	+
18	46,XX,der(5;13)(p10;q10),add(17)(p13),+19,-20 [10]/46,XX [1]	+	+
19	53-57,XY,+1,add(1)(q23),dic(1;11)(q11;p11),+2,add(2)(q37),add(2)(q21),der(2;7)(p10;q10),add(7)(q11),+8,+9,+10,+11,+13,+15,-17,+20,add(21)(q22),+22,+3mar [cp20]	+	+
20	43-45,XY,add(1)(q10),add(4)(p12),-5,add(6)(q13),-7,add(9)(q34),add(12)(p11),-15,-17,-20,+2-4mar [9]	+	+
21	44,XX,-2,-5,+8,-10,-12,der(14)t(14;15),(q22;q15),-15,-17,add(22)(q11),+3mar [7]/45,XX,add(2)(q11),-5,+8,-10,-12,der(14)t(14;15)(q22;q15),-15,-17,add(22)(q11),+3mar [3]	+	+
22	45,XY,-9,add(17)(p13),del(20)(q11) [11]	+	+
23	43-49,XY,der(3),del(5)(q),-14,-15,-17,-18,-20,+21,+1-5mar [cp8]	+	+
24	46,XX [1]/45,X,-X,add(5)(q11),-17,add(18)(q?22),-21,+mar,+mar [22]	+	+
25	46,XY,inv(3)(q21q26.2),del(6)(q12q23),dup(7)(q11q21),del(7)(q22),-17,+mar [7]/46,XY[4]	+	+

Abbreviations: Pat, patient; CK, complex karyotype; MK, monosomal karyotype; n.a., not applicable; n.d., not determined

* Patient numbers correspond to numbers in Figure 1

Supplemental Table S2. Gene mutations identified by panel sequencing

Pat	Gene	Chr	Nucleotide change	AA change	VAF	Major clone (M) vs minor subclones (m)
16	TP53	17p13.1	c.365_366delTG	p.V122fs*26	44%	M
16	TP53	17p13.1	c.734G>C	p.G245A	37%	M
16	PTPN11	12q24.13	c.218C>T	p.T73I	32%	M
17	TP53	17p13.1	c.524G>A	p.R175H	38%	M
18	TP53	17p13.1	c.707A>G	p.Y236C	81%	M
18	DNMT3A	2p23.3	c.1220T>C	p.I407T	45%	M
18	KIT	4q12	c.2447A>T	p.D816V	17%	m
18	PTPN11	12q24.13	c.182A>G	p.D61G	11%	m
19	TP53	17p13.1	c.747G>C	p.R249S	57%	M
19	ABL1	9q34.12	c.613G>A	p.V205I	44%	M
20	JAK2	9p24.1	c.1624_1629delAATGAA	p.N542_E543del	55%	M
20	TP53	17p13.1	c.524G>A	p.R175H	50%	M
21	BCOR	Xp11.4	c.4537C>T	p.R1513*	87%	M
21	TP53	17p13.1	c.308dupA	p.Y103*	75%	M
22	TP53	17p13.1	c.535C>T	p.H179Y	21%	M
22	DNMT3A	2p23.3	c.2645G>A	p.R882H	17%	M
22	FLT3	13q12.2	c.2503G>T	p.D835Y	12%	M
23	TP53	17p13.1	c.1018delA	p.M340fs*5	21%	M
24	EZH2	7q36.1	c.1355dupA	p.Y452*	81%	M
24	ASXL1	20q11.21	c.1934dupG	p.G646Wfs*12	36%	M
25	ZRSR2	Xp22.2	c.515dupG	p.C172Wfs*6	89%	M
25	RUNX1	21q22.12	c.328A>C	p.K110Q	52%	M
25	ASXL1	20q11.21	c.2302C>T	p.Q768*	51%	M
25	ASXL1	20q11.21	c.3306G>T	p.E1102D	51%	M
25	EZH2	7q36.1	c.907+2T>C	splicing	49%	M
25	RUNX1	21q22.12	c.496C>G	p.R166G	45%	M
25	FLT3	13q12.2	c.2533A>G	p.R845G	25%	m
25	FLT3	13q12.2	c.1775T>A	p.V592D	6%	m
25	EZH2	7q36.1	c.1307A>G	p.E436G	46%	M
26	EZH2	7q36.1	c.1583G>C	p.C528S	85%	M
26	RUNX1	21q22.12	c.482T>A	p.L161H	47%	M
26	KRAS	12p12.1	c.34G>A	p.G12S	46%	M
26	ASXL1	20q11.21	c.2077C>T	p.R693*	43%	M
27	SRSF2	17q25.1	c.283C>G	p.P95A	47%	M
27	EZH2	7q36.1	c.2080C>T	p.H694Y	46%	M
27	EZH2	7q36.1	c.73C>T	p.R25*	46%	M
27	IDH1	2q34	c.394C>T	p.R132C	45%	M
27	RUNX1	21q22.12	c.500G>C	p.S167T	44%	M
27	ASXL1	20q11.21	c.1934dupG	p.G646Wfs*12	33%	m
28	RUNX1	21q22.12	c.965C>G	p.S322*	40%	M
28	BCOR	Xp11.4	c.2893A>T	p.R965*	38%	M
28	STAG2	Xq25	c.1876dupA	p.T626Nfs*9	38%	M
28	GATA2	3q21.3	c.253_256dupTGCC	p.R86Lfs*100	35%	M
28	PHF6	Xq26.2	c.821G>A	p.R274Q	33%	M
28	ASXL1	20q11.21	c.1934dupG	p.G646Wfs*12	32%	M
29	TET2	4q24	c.5122delA	p.S1708fs*11	47%	M
29	KRAS	12p12.1	c.34G>C	p.G12R	39%	M
29	ASXL1	20q11.21	c.1934dupG	p.G646Wfs*12	36%	M
30	STAG2	Xq25	c.646C>T	p.R216*	82%	M
30	IDH2	15q26.1	c.419G>A	p.R140Q	45%	M
30	ASXL1	20q11.21	c.2278C>T	p.Q760*	41%	M
31	SRSF2	17q25.1	c.284C>T	p.P95L	54%	M
31	IDH1	2q34	c.394C>T	p.R132C	49%	M
31	RUNX1	21q22.12	c.610C>T	p.R204*	45%	M
31	ASXL1	20q11.21	c.1900_1922delAGAGAGGCGGCCACCACTGCCAT	p.E635Rfs*15	42%	M
31	WT1	11p13	c.1390G>A	p.D464N	30%	m
32	RUNX1	21q22.12	c.318G>C	p.W106C	81%	M
32	IDH2	15q26.1	c.419G>A	p.R140Q	45%	M
32	SRSF2	17q25.1	c.284C>G	p.P95R	43%	M
32	FLT3	13q12.2	c.1727T>C	p.L576P	33%	m
32	ASXL1	20q11.21	c.1934dupG	p.G646Wfs*12	32%	m
33	CBL	11q23.3	c.1268T>A	p.I423N	92%	M
33	SRSF2	17q25.1	c.284C>A	p.P95H	48%	M
33	TET2	4q24	c.1123G>T	p.E375*	45%	M
33	TET2	4q24	c.3732_3733delCT	p.Y1245Lfs*22	45%	M
33	RAD21	8q24.11	c.1681C>T	p.Q561*	43%	M
33	RUNX1	21q22.12	c.1090_1103delATCGGCATCGGCAT	p.I364Vfs*231	43%	M
33	ASXL1	20q11.21	c.1772dupA	p.Y591*	38%	M
34	SRSF2	17q25.1	c.284C>T	p.P95L	41%	n.a.
34	IDH1	2q34	c.394C>A	p.R132S	36%	n.a.
34	ASXL1	20q11.21	c.1934dupG	p.G646Wfs*12	28%	n.a.
35	SRSF2	17q25.1	c.284C>A	p.P95H	54%	n.a.
35	IDH2	15q26.1	c.419G>A	p.R140Q	51%	n.a.
35	ASXL1	20q11.21	c.2708C>A	p.S903*	49%	n.a.
36	SRSF2	17q25.1	c.284C>T	p.P95L	49%	n.a.
36	ASXL1	20q11.21	c.2290delC	p.L764Yfs*8	46%	n.a.

36	GATA2	3q21.3	c.1163_1168delTGAAGA	p.M388_K389del	46%	n.a.
36	NRAS	1p13.2	c.35G>A	p.G12D	31%	n.a.
36	NRAS	1p13.2	c.182A>G	p.Q61R	16%	n.a.
36	WT1	11p13	c.1137_1141dupACGGT	p.S381Yfs*70	8%	n.a.
37	EZH2	7q36.1	c.2051G>A	p.R684H	83%	M
37	DNMT3A	2p23.3	c.1240T>G	p.F414V	48%	M
37	TET2	4q24	c.679G>T	p.E227*	46%	M
37	SF3B1	2q33.1	c.1986C>A	p.H662Q	41%	M
37	FLT3	13q12.2	c.2503G>T	p.D835Y	23%	m
37	CBL	11q23.3	c.1111T>G	p.Y371D	7%	m
38	BCOR	Xp11.4	c.4266dupT	p.I1423Yfs*4	32%	n.a.
38	STAG2	Xq25	c.3085C>T	p.Q1029*	26%	n.a.
38	DNMT3A	2p23.3	c.2225G>C	p.R742P	17%	n.a.
38	CEBPA	19q13.11	c.868G>T	p.E290*	16%	n.a.
38	SF3B1	2q33.1	c.1874G>T	p.R625L	16%	n.a.
38	DNMT3A	2p23.3	c.490G>A	p.E164K	11%	n.a.
38	RUNX1	21q22.12	c.772G>A	p.A258T	7%	n.a.
39	DNMT3A	2p23.3	c.2635A>G	p.N879D	34%	M
39	PHF6	Xq26.2	c.946A>T	p.N316Y	32%	M
39	STAG2	Xq25	c.328C>T	p.R110*	26%	M
39	IDH2	15q26.1	c.419G>A	p.R140Q	25%	M
39	BCORL1	Xq26.1	c.4685G>T	p.S1562I	22%	M
39	BCOR	Xp11.4	c.1005dupC	p.S336fs*45	19%	M
40	BCORL1	Xq26.1	c.2715_2716insCC	p.K906Pfs*20	30%	M
40	IKZF1	7p12.2	c.482T>C	p.L161P	28%	M
41	NPM1-B	5q35.1	c.863_864insCATG	p.W288fs*12	32%	M
41	KDM6A	Xp11.3	c.1063C>T	R355*	27%	M
41	FLT3	13q12.2	c.2516A>C	p.D839A	23%	M
41	IDH2	15q26.1	c.419G>A	p.R140Q	14%	M
41	FLT3	13q12.2	c.2533A>G	p.R845G	10%	M
42	DNMT3A	2p23.3	c.1019delG	p.C340Lfs*5	40%	M
43	DNMT3A	2p23.3	c.2645G>C	p.R882P	45%	M
43	FLT3	13q12.2	c.1734_1781dupGGTACAGGTGACCGGCTCCTCAG ATAATGAGTACTTCTACGTTGATTT	p.F594insLVQVTG SSDNEYFYVDF	14%	m
44	STAG2	Xq25	c.328C>T	p.R110*	96%	M
44	CBL	11q23.3	c.1259G>A	p.R420Q	77%	M
44	DNMT3A	2p23.3	c.1627G>T	p.G543C	50%	M
44	IDH1	2q34	c.394C>T	p.R132C	49%	M
45	TET2	4q24	c.736dupA	p.T246Nfs*8	94%	M
45	SRSF2	17q25.1	c.284C>A	p.P95H	57%	M
45	NPM1	5q35.1	c.860_863dupTCTG	p.W288Cfs*12	47%	M
46	TET2	4q24	c.3866G>A	p.C1289Y	83%	M
46	JAK2	9p24.1	c.1849G>T	p.V617F	35%	M
47	TET2	4q24	c.3782G>A	p.R1261H	90%	M
47	SRSF2	17q25.1	c.284C>T	p.P95L	53%	M
47	RUNX1	21q22.12	c.319C>T	p.R107C	43%	M
47	RUNX1	21q22.12	c.494_497delinsTCTGT	p.G165Vfs*48	39%	M
47	PTPN11	12q24.13	c.181G>T	p.D61Y	11%	m
47	PTPN11	12q24.13	c.226G>A	p.E76K	8%	m
47	PTPN11	12q24.13	c.172A>T	p.N58Y	8%	m
48	TET2	4q24	c.2716_2717delAT	p.M906Vfs*17	46%	n.a.
48	NPM1	5q35.1	c.860_863dupTCTG	p.W288Cfs*12	35%	n.a.
49	CUX1	7q22.1	c.4035G>C	p.E1345D	48%	n.a.
49	TET2	4q24	c.992delT	p.I331Nfs*16	44%	n.a.
49	NPM1-A	5q35.1	c.860_863dupTCTG	p.W288Cfs*12	38%	n.a.
49	PHF6	Xq26.2	c.454G>T	p.E152*	20%	n.a.
49	GATA2	3q21.3	c.1163T>C	p.M388T	18%	n.a.
49	PHF6	Xq26.2	c.480_481delinsTT	p.K160_K161delins N*	8%	n.a.
50	IDH2	15q26.1	c.515G>A	p.R172K	32%	n.a.
50	RUNX1	21q22.12	c.568_569insGCCTC	p.H190Rfs*23	18%	n.a.
51	SRSF2	17q25.1	c.284_307delCCCCGGACTCACACCACAGCCGCC	p.95_103delPPDSH HSRR	35%	M
52	SRSF2	17q25.1	c.284_307delCCCCGGACTCACACCACAGCCGCC	p.P95_R102del	15%	n.a.
52	CBL	11q23.3	c.1100A>C	p.Q367P	12%	n.a.
52	GATA2	3q21.3	c.488C>T	p.A163V	11%	n.a.
52	CSF3R	1p34.3	c.2395T>C	p.S799P	10%	n.a.
53	RUNX1	21q22.12	c.472T>G	p.F158V	44%	M
53	SF3B1	2q33.1	c.1986C>A	p.H662Q	37%	M
53	RUNX1	21q22.12	c.523_528dupCTGACC	p.L175_T176dup	30%	M
53	FLT3	13q12.2	c.2503G>T	p.D835Y	18%	m
53	CBL	11q23.3	c.1151G>A	p.C384Y	8%	m
54	SF3B1	2q33.1	c.1997A>C	p.K666T	47%	n.a.
54	FLT3	13q12.2	c.1784_1804dupGAGAATATGAATATGATCTCA	p.K602insREYEYD LK	24%	n.a.
55	ZRSR2	Xp22.2	c.515G>A	p.C172Y	8%	M
56	RAD21	8q24.11	c.2_3insCCGAGAG	p.M1Ifs*12	13%	M
57	FLT3	13q12.2	c.2503G>A	p.D835N	30%	M
58	n.a.	n.a.	n.a.	n.a.	n.a.	n.a.
59	n.a.	n.a.	n.a.	n.a.	n.a.	n.a.
60	n.a.	n.a.	n.a.	n.a.	n.a.	n.a.

Minor subclone >20% smaller than major clone; n.a., not available

Supplemental Table S3. Response rates and overall survival according to the mutation status as indicated.

	**CR/PR/ALE n (%)**	**CR/PR/ALE vs SD/PD/ED *P-value***	**Median OS (months)**	***P-value***
**>3 mutated genes** (n=18) *	9 (53%)	-	3.6	-
≤3 mutated genes (n=27)	12 (44%)	0.76	4.3	0.74
**Minor subclone** (n=9)	5 (56%)	-	2.9	-
No minor subclone (n=24)	10 (42%)	0.70	5.0	0.05
***TP53* mut or minor subclone** (n=16)	8 (50%)	-	2.8	-
TP53 wt and no minor subclone (n=17)	7 (41%)	0.73	5.6	0.01

* In 1 patient best response was not evaluable
